# A research agenda for open energy science: Opportunities and perspectives of the F1000Research Energy Gateway

**DOI:** 10.12688/f1000research.124267.1

**Published:** 2022-08-04

**Authors:** Daniel Huppmann, Jethro Browell, Benedetto Nastasi, Zita Vale, Diana Süsser

**Affiliations:** 1 Energy, Climate and Environment Program (ECE), International Institute for Applied Systems Analysis (IIASA), Laxenburg, Austria; 2 School of Mathematics & Statistics, University of Glasgow, Glasgow, UK; 3 Department of Planning Design & Technology of Architecture, Sapienza University of Rome, Rome, Italy; 4 Polytechnic of Porto, Porto, Portugal; 5 Institute for Advanced Sustainability Studies (IASS), Potsdam, Germany

**Keywords:** Energy transition, Open Science, FAIR Data

## Abstract

Energy enables the functioning of modern society. However, humanity’s reliance on fossil fuels since the industrial revolution has contributed to many societal problems including climate change, environmental degradation and pollution, and the transition to a renewable and carbon-free energy system is one of the grand challenges for the 21st century. The aim of this editorial is to outline the importance of a fast and transparent sharing of energy research and discuss key themes of the Energy Gateway of F1000Research.

Energy enables the functioning of modern society. However, humanity’s reliance on fossil fuels since the industrial revolution has contributed to many societal problems including climate change, environmental degradation and pollution ( IPCC, 2022a). As a consequence, the transition to a renewable and carbon-free energy system is one of the grand challenges for the 21st century. The recently published IPCC AR6 WG3 report ( IPCC, 2022b) highlighted the importance of the energy transition to mitigate climate change as well as its co-benefits and synergies across the Sustainable Development Goals. For example, model-based analysis and quantitative assessments such as the scenario ensemble used in the IPCC reports can inform policy-makers and society at large about synergies and trade-offs between various strategies and pathways towards climate neutrality ( Süsser et al., 2022; Guivarch et al., 2022). The aim of this editorial is to outline the importance of a fast and transparent sharing of energy research and discuss key themes of the Energy Gateway of F1000Research.

## Open Science is better research

Researchers have to meet new expectations for transparency, intelligibility and reproducibility of their work to allow scrutiny and foster credibility of the findings. Open science requires a trifecta of 1) data that is findable, accessible, interoperable and reusable (known as the FAIR principles, Wilkinson, 2016), 2) open-source scientific software and tools, and 3) open-access manuscripts to describe data, methods and insights. Each of these components of research has very different "natural" ways of dissemination: For research data, figshare, Zenodo or an institutional data repository are the prefered methods to share and archive datasets in line with the FAIR principles of data management and stewardship. For scientific software, GitHub, BitBucket and similar services can facilitate the adoption of best-practice collaborative scientific-software development ( Wilson et al., 2016). It is quickly becoming the norm to release scripts and tools under open-source licenses to enable others to inspect, reuse and further develop the computational foundation of much state-of-the-art research. The phrase “available upon reasonable request” is becoming a red flag in the scientific community.

For scientific manuscripts, peer review is rightly considered as the gold standard, and open access to manuscripts is quickly becoming a requirement by funding agencies. This is a substantial improvement compared to pay-walls, which often exclude researchers from small institutions or lower-income countries. However, processing fees for publishing an accepted manuscript as an open-access resource are often very steep, and the peer-review process can cause substantial delay, hindering an effective and timely dissemination of new research methods or insights. Furthermore, anonymous and closed peer-review leaves readers unable to know the level of scrutiny a publication has received.

## A publication strategy to foster Open Science and rapid dissemination

Current practice in scientific research is often to treat the research data, the scientific software or analysis scripts, and the description of methods and findings as a “package” which is only released in its entirety at the end of the peer-review process when the manuscript is published. Given the substantial delay often caused by peer review, this can hinder the quick adoption of new methods or a timely re-examination of datasets for further insights. Also, researchers often feel pressured to hold back minor improvements until a sufficiently “publishable” amount of work has been done for submission to a top-tier journal.

The publication strategy facilitated by F1000Research can overcome several shortcomings of the current academic workflow. First, due to the post-publication, open peer review, manuscripts can be shared with a clear attribution record much earlier, so there are fewer incentives to hold back data or scientific software/methods until final acceptance of a manuscript. Second, providing an easy way to submit revisions or updates to already-published manuscripts facilitates a more continuous approach to research. Third, the broad scope of F1000Research allows publishing the different components of science (data, scientific software, research manuscripts) individually, thereby speeding up the pace of knowledge creation and sharing. Finally, open peer review increases transparency for readers, authors and reviewers, and increases trust in the publishing process.

## Outlook for the Energy Gateway

There are numerous technologies that have the potential to contribute to the decarbonization of the energy system including renewable electricity and green hydrogen. Demand flexibility and different forms of delivering it by means of demand response ( Silva et al., 2022) can provide system reliability previously offered by dispatchable fossil power generation. New operational practices as well as batteries and other forms of energy storage can balance supply and demand over time enabling the path for almost 100% renewable energy systems. New materials and behavioral changes can facilitate energy efficiency and sufficiency. Effective use of distributed energy resources in the scope of energy communities or by individuals and innovative energy transaction models and market-driven approaches ( Lezama et al., 2018) will be important drivers towards a decarbonized energy system. Economics, justice, and equity of the transition must be balanced to ensure socially and politically feasible transitions. Research on all these topics fits well within the scope of the Energy Gateway.

We see particular relevance for a new journal of energy research for several reasons:

First, following high-level events like the UN Climate Change Conference COP26 in November 2021, there is a need for fast-turnaround, policy-relevant publications. For example, the work by Meinshausen et al. (2022) was widely discussed at COP26, but the scientific publication was only published 5 months after the event - and almost half of that time period was between acceptance and publication. A faster publication and review process can facilitate a better dialogue between researchers and policy-makers ( Süsser et al., 2021).

Second, data collections related to energy technologies and policy, evaluation of Nationally Determined Contributions (NDCs), or collaborative, community-driven efforts to compile statistics that serve as alternatives to the IEA’s World Energy Outlook or BP’s Statistical Review of World Energy are crucial for policy-relevant and timely research. However, scientific manuscripts describing these data and underlying methodology used for data collection and analysis remain mainly unpublished because they are often not seen as sufficiently novel by top-tier journals. An example of such work is the Public Utility Data Library (PUDL). A few isolated initiatives have started in recent years, such as the special issues of some journals ( Nastasi et al. 2020, Nastasi et al. 2021) but they do not yet yield a comprehensive publishing strategy in the field. Also, going beyond descriptive data collections, we see a need for benchmark data that can be used to train machine learning algorithms applied to energy research. The IEEE plays that role for different power systems topics providing publicly available datasets, for instance in the IEEE DataPort and in the Open Datasets with real, measured data provided by the IEEE Power Engineering Society Intelligent Systems Subcommittee. There are numerous fields that can benefit from well-crafted, standardized datasets that facilitate comparison and evaluation of alternative computational methods. Here, we hope that the Energy Gateway can establish itself as a true gateway for energy statistics and data as well as their analysis and interpretation.

Third, we see that energy research must place even higher emphasis on social science and humanities (SSH) related to the energy transition, because the required changes are “a monumental cultural and political challenge” ( Stirling, 2014). Societies are not only affected by the transition, but can also shape it by driving it forward - or in contrast, a lack of awareness and limited social acceptability can substantially delay the necessary changes. Researchers have outlined diverse directions for SSH research, including research on deep transformations, influence of cultural and geographic diversity, energy governance and value-based goals instead of instrumental acceptance ( Krupnik et al., 2022). These and other directions and themes require “open spaces” for critical discussion on strategies and pathways of energy transitions. In this Energy Gateway, we aim to provide space for publications of various SSH research fields and joint research work from different knowledge communities. We therefore call for SSH research contributions from across the globe to help to close the gap between frontrunners and laggards of the energy transition ( Quitzow et al., 2021) and to unfold the transition globally. Moreover, we call for SSH research to improve our understanding of societal values, preferences, concerns and motivations across countries and regions, for example, to enable the design of inclusive energy policies and better linking of SSH with quantitative energy modeling.

In many ways, the agenda for Energy Gateways confirms the importance of open science, mutual consideration of different research fields and bridging of different energy research communities for rapid and deep decarbonization. We believe that the Energy Gateway can serve as a useful resource and publication outlet for this kind of research, contributing to more open and collaborative research and helping to guide society on its path towards a carbon-free and sustainable energy supply.

## Data availability

No data is associated with this article.
